# Development and cross-validation of a novel multi-omic assay to assess locoregional recurrence risk and adjuvant therapy benefit in early-stage hormone receptor positive invasive breast cancer patients

**DOI:** 10.1186/s13058-026-02237-4

**Published:** 2026-02-18

**Authors:** Troy Bremer, Karuna Mittal, Chirag Shah, Frank Vicini, Naamit K. Gerber, Melissa Krystel-Whittemore, Clayton C. Yates, Balasubramanyam Karanam, Walter Bell, Samuel G. Borak, Charles E. Cox, Abigail Beard, Geza Acs, Vincent Reid, Zahraa Al-Hilli, Steven C. Shivers, Mark Mentrikoski, David Dabbs, Jess Savala, Pat W. Whitworth, Charlotta Wadsten

**Affiliations:** 1 PreludeDx, Laguna Hills, CA USA; 2 Allegheny Health Network, Pittsburgh, PA USA; 3 Michigan Healthcare Professionals, Farmington Hills, MI USA; 4 Laura and Isaac Perlmutter Cancer Center, New York, NY USA; 5 NYU Grossman School of Medicine, New York, NY USA; 6 Johns Hopkins School of Medicine, Baltimore, MD USA; 7 Tuskegee University, Tuskegee, AL USA; 8 Baptist Health, University of Alabama Birmingham, Montgomery, AL USA; 9 University of South Florida, Morsani College of Medicine, Tampa, FL USA; 10 AdventHealth Tampa, Tampa, FL USA; 11 Hall Perrine Cancer Center, Mercy Medical Center, Cedar Rapids, IA USA; 12 Integrated Surgical Institute, Cleveland Clinic, Cleveland, OH USA; 13 Sundsvall Hospital, Sundsvall, Sweden

## Abstract

**Purpose:**

Breast cancer management is shifting towards personalized treatment regimens, particularly for early-stage, hormone receptor positive (HR+) invasive breast cancer (IBC) patients following breast conserving surgery (BCS) where locoregional recurrence (LRR) rates are low. A critical unmet need is the development of tools that can both improve prognostic risk assessment and identify which patients are likely to benefit or not benefit from adjuvant radiation therapy (RT). Herein we developed and cross validated a novel multi-omic assay to assess LRR risk and expected RT benefit for early-stage HR+/HER2-negative IBCs.

**Methods:**

A retrospective multi-institutional cohort of 922 patients (T1-2, N0-1, HR+, HER2-) treated with definitive breast conserving surgery (BCS) with or without adjuvant treatment was used to develop and cross-validate a test to predict IBC LRR after BCS ± RT. Treatment assignment was not randomized. The test integrated NGS and proteomic assay data using two biosignatures to generate results: a Decision Score (DS) to predict 10-year LRR prognosis and a radiation resistance index (RRI) to predict differential RT effect on LRR. Associations between DS and RRI with LRR risk and RT interaction were tested using multivariable Cox models.

**Results:**

Increasing continuous DS was associated with increasing LRR risk (HR 3.4 per 5 units; *p*<.001, *n* = 922) after adjusting for clinicopathologic risk factors, while RT was associated with reduced LRR risk (HR 0.2; *p* < .001). Increasing continuous RRI was associated with increasing LRR risk for patients treated with RT (HR = 3.1 per 5 units; RT: RRI p_interaction_ = 0.002). Biosignature utility was demonstrated for categorical risk groups, which were also associated with differential RT benefit. DS Elevated Risk patients (DS > 5) had higher LRR risk without RT (HR = 4.8; *p* = .0014) with corresponding 10-year risks of 24% in DS Elevated Risk versus 7% in DS Low Risk (DS ≤ 5). In DS Low Risk patients, a statistically significant reduction in LRR risk (HR = 1.0, *p* = .96) was not observed. However, in the DS Elevated Risk group, RT was associated with decreased LRR risk (HR = 0.4, *p* = .0097) for patients with a lower radio resistance index (DS > 5, RRI ≤ 5), whereas RT was not associated with a statistically significant reduction in LRR risk (HR = 0.8, *p* = .51) for patients with a higher radio resistance index (DS > 5, RRI > 5).

**Conclusions:**

In this large retrospective, non- randomized multi-institutional cohort, the novel multi-omic biosignature was associated with lower and higher LRR risk after BCS and associated with statistically significant differential RT effect for LRR risk reduction. Clinically meaningful risk groups that were associated with differential RT benefit were identified using the biosignature, supporting its potential utility in the assessment of the LRR risk in early-stage HR+/HER2-negative IBC. Further clinical validation studies are in process, and prospective validation studies are being planned to establish the role of the test in clinical practice.

**Supplementary Information:**

The online version contains supplementary material available at 10.1186/s13058-026-02237-4.

## Introduction

Adjuvant radiation therapy (RT) has represented a standard component of breast conservation therapy (BCT) for treating invasive breast cancer, demonstrating a reduction in invasive local recurrence and improvement in breast cancer mortality in patients with varied tumor types and ages in randomized clinical trials [1, 2]. However, these trials also demonstrated that only a subset of patients benefited from RT after BCS to prevent subsequent breast cancer events, and given the associated toxicities, attempts have been made for decades to omit RT following breast conserving surgery (BCS) based on clinicopathologic based risk assessment [3–5]. For example, the CALGB 9343 trial evaluated women aged 70 years or older with T1N0, estrogen receptor positive breast cancers with negative margins following BCS. Patients were treated with endocrine therapy and randomized to RT or no RT and at 10 years the omission of RT was associated with increased rates of local recurrence (10% vs. 2%) with no impact on survival [6]. Similarly, the PRIME II trial evaluated omission of RT in women 65 years or older with T1-2 (≤ 3 cm), N0, hormone receptor positive, human epidermal growth factor receptor 2 negative (HR+/HER2-) invasive breast cancer following BCS. Patients were randomized to endocrine therapy with or without RT and at 10 years, consistent with the CALGB 9343 trial, omission of RT was associated with an increased risk of local recurrence (10% vs. 1%) with no impact on survival [7]. These findings highlight the limitations of clinical and pathological criteria to accurately identify patients in whom omission of radiation does not impact survival but increases the risk of locoregional recurrence, leading to variation in de-escalation practices across the United States.

Currently, the landscape of locoregional therapy for early-stage breast cancer continues to evolve with a focus on individualized risk stratification to allow for informed decision making for patients with early-stage, HR+/HER2- invasive breast cancer. To date, studies have been published attempting to integrate tumor molecular biology in this effort, but a key challenge is identifying assays that are not only prognostic of invasive locoregional recurrence (LRR) but also predictive for RT benefit after adjusting for clinicopathologic risk factors [8, 9]. Similar to how the DCISionRT(^TM^) test has provided an integrated approach for DCIS patients by identifying both prognosis for in-breast recurrence and RT benefit, there is critical need for a comparable tool in invasive disease. To this end we developed and cross-validated a novel multi-omic assay designed specifically to assess invasive LRR risk as well as the differential RT effect on LRR risk in early-stage HR+/HER2-negative breast cancer patients treated with BCS +/- ET.

## Patients and methods

This multi-institutional study was conducted on archived tissue samples from four centers: Uppsala University Hospital and Västmanland County Hospital, Sweden (UUH), the Baptist Hospital, Alabama (BAL), the University of South Florida (USF), and New York University (NYU), in collaboration with the study sponsor, PreludeDx. Patients were included between 1986 and 2004 at UUH, 1992 and 2009 at BAL, 2012 and 2022 at USF, and 2013 and 2021 at NYU. All patients underwent BCS with or without RT and some were treated with ET or chemotherapy (CT) (Supplemental Table 1). Adjuvant treatment decisions were neither randomized nor strictly rules based. Exclusion criteria included mastectomy, Tis, T3 or T4 disease, N2 or N3 disease, hormone receptor negative disease, HER2-positive disease or metastatic disease. Initially, 1249 patients were identified who underwent BCS, with 922 patients meeting inclusion and exclusion criteria with FFPE tissue blocks or FFPE tissue microarray biopsies and pathology reports available, and with 757 patients of age 50 years or older, (Supplemental Fig. 1). As all of the eligible patients were included, no separate power analysis was conducted. The study was conducted with the appropriate institutional approvals and in accordance with recognized ethical guidelines and principles from the WMA Declaration of Helsinki for medical research involving human subjects.

### Biological signature (AidaBREAST(^TM^), PreludeDx, Laguna Hills, CA) development

The multi-omic test integrates protein expression (multiplex-immunofluorescence (MIF) and spatial biology) and targeted mRNA next generation sequencing (Supplemental Information) [10–12] to determine the prognosis for invasive LRR after BCS and expected benefit from RT. No clinicopathologic features were included in the biosignatures, and the model outputs are independent of clinicopathologic features. Using standardized protocols adapted for MIF, spatial biology, and RNA seq workflows, FFPE was assayed in a centralized lab and two biosignatures were calculated (Supplemental Information) [10–12]. The prognosis for LRR after BCS was determined using a Decision Score (DS) and a radiation resistance index (RRI) was used to assess the efficacy of RT. These two biosignatures were constructed using protein expression of selected markers and Gene Set Enrichment Analysis (GSEA) of mRNA expression of gene sets, representing key signaling pathways (Supplemental Tables 2–4). The biosignatures were parameterized and tested using 500-fold cross-validation stratified by study site. For each fold, 70% of the eligible subjects were randomly assigned to the training set and 30% to the validation set. The median value of the validation results for each patient was used to calculate consensus continuous scores that were scaled from 0 to 10 points. For the purpose of summarizing test performance using survival analyses, the continuous Decision Score biosignature and Radiation Resistance Index (RRI) biosignature were additionally presented using categorical groupings as secondary analyses to facilitate clinical interpretation. The DS categorical threshold was selected with the goal of maximizing the hazard ratio between low and high DS LRR risk groups in patients treated without RT, where patients with a score greater than the threshold (DS = 5.0) belonged to the Elevated Risk group (DS > 5). For RRI threshold used was selected by machine learning with the goal of identifying patients with lower (RRI > 5) and higher differential RT effect (RRI < = 5). The DS and RRI results together identify an elevated LRR risk group expected to have a lower RT effect (DS > 5 and RRI > 5) and a higher RT effect (DS > 5 and RRI ≤ 5) for LRR risk. Alternative RRI thresholds for characterizing LRR risk and RT benefit were also evaluated.

### Statistical methods

The primary end point of this study was invasive locoregional recurrence (LRR) free survival. LRR was histologically defined as confirmed invasive disease in the ipsilateral breast or regional lymph nodes. Details regarding event definitions, censoring rules, and analyses for calculating invasive LRR, distant metastasis (DM), and contralateral invasive breast events are provided in Supplemental Information.

Descriptive statistics summarized clinicopathologic and treatment characteristics. Differences in clinicopathologic and treatment characteristics by treatment and study sites were assessed using Chi-square testing, and corresponding *p*-values were reported. Multivariable Cox proportional hazards analyses for LRR risk were performed with covariates for treatment and clinicopathologic factors and with the continuous biosignature scores DS and RRI. The interaction between RRI and RT was assessed along with DS, and treatment by RT and ET as independent factors, and the multivariable analyses were stratified by chemotherapy treatment (CT). A corresponding analysis was completed for women diagnosed at 50 years of age and older. Ninety Five (95%) confidence intervals for Cox proportional hazards and Kaplan Meier survival analyses were calculated using 1000-fold stratified bootstrap analysis (Supplemental Methods). For Kaplan–Meier survival analyses, 95% confidence intervals were obtained using a 1000-fold stratified nonparametric bootstrap (resampling within study centers) and BCa intervals were reported. Nested Cox proportional hazards models (CP and treatment vs. CP and treatment plus biosignatures) were compared using a likelihood ratio test. The performance of the test was further evaluated using propensity score adjustment to account for the impact of non-randomized RT treatment. The propensity score was calculated by logistic regression modeling for RT (yes/no) using a generalized linear model (GLM) with clinicopathologic factors and treatment (ET and CT). LRR risk differences between risk groups were evaluated using the log-rank test and corresponding *p*-values were reported. All statistical analysis were performed using R software, version 4. 3.1. See supplemental information for further details.

## Results

### Study cohort clinicopathology, treatment characteristics, and outcomes

A total of 922 women underwent definitive BCS for HR+/HER2- invasive breast cancer at four study centers and had FFPE tissue with treatment and outcome data, as shown in the consort diagram (Supplemental Fig. 1). Of the 922 eligible patients, 736 (80%) were treated with RT and 407 (44%) were treated with ET (Table 1). Young women (age < 50) and patients with node positive disease were more likely treated with RT. In univariable analysis, RT, ET and CT were not associated with a statistically significant reduction in LRR Risk (note: 84% of patients receiving ET received RT, and 90% of patients receiving CT received RT), but young age (< 50) was associated with increased LRR risk (Table 2). The 10-year LRR risk was 13% vs. 8% for women who were treated without RT versus with RT (± ET, ±CT) independent of the biosignature, see (Supplemental Table 5). The 10-year contralateral breast event risk was 4% for those patients prescribed ET (*n* = 407) versus 7% for those not prescribed ET (*n* = 515). The 10-year distant metastasis rate was 5% for those prescribed ET and not prescribed ET. There were few regional events (< 1%) in the overall eligible cohort (Supplemental Table 5).

There were 757 (82%) patients of age 50 years or older with a 65-year median age (Supplemental Table 6). Ipsilateral locoregional, contralateral, and distant metastatic risks were similar to the overall population (Supplemental Table 7). In univariable analysis, RT decreased LRR risk (HR = 0.6; 95% CI 0.4 to 0.9; *p* =.037), while ET and CT were not associated with a statistically significant reduction in LRR Risk (Supplemental Table 8) (note: in women > 50, 80% of patients receiving ET received RT, and 89% of patients receiving CT received RT).

## Locoregional recurrence risk—continuous test results

**Fig. 1:**
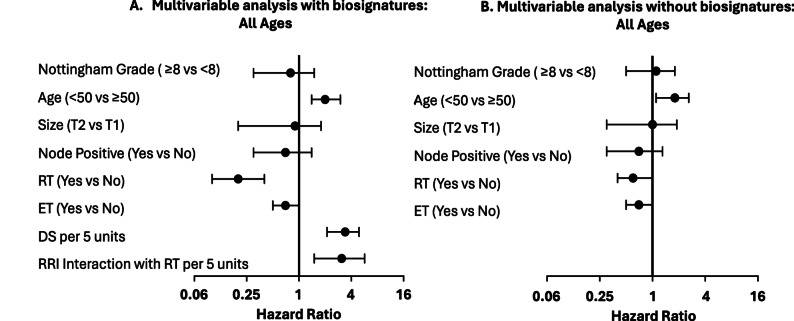
Forest plot of multivariable analysis of clinicopathology and biosignatures in women of all ages included in study. **A** Biosignature and treatment adjusted for the clinicopathology factors. **B** Clinicopathology factors and treatment alone. The biosignature added information not available from CP factors and treatment alone (*p*<.001)

The test reported Decision Score (DS) and Radio-resistance Index (RRI), each on a 10-point scale, to assess prognosis for LRR and expected RT benefit. Increasing continuous DS was associated with higher LRR risk (adjusted HR = 3.3 per 5 units; 95% CI 2.1–4.8; *p*<.001), Fig. 1 and Supplemental Table 7. RT was associated with lower LRR risk (adjusted HR 0.2; 95% CI 0.1–0.4; *p*<.001), but RRI had a significant interaction with RT (p_interaction_ = .002). Among patients treated with RT, increasing RRI was associated with increasing LRR risk (adjusted HR = 3.1 per 5 units; 95% CI 1.5–5.7). Endocrine therapy was not associated with a significant decrease in LRR risk but trended toward a lower LRR risk (adjusted HR = 0.7; 95% CI 0.5–1.0; *p* = .14). Young age was associated with LRR risk, no other clinicopathological risk factors had a statistically significant association with LRR. The biosignature results provided information that was not available from clinicopathology factors and treatment alone based on log-likelihood testing (*p*<.001), see Supplemental Table 9. Consequently, after accounting for DS and RRI in the multivariable analysis, the RT effect was more pronounced, with an adjusted HR = 0.2 (95%CI 0.1–0.4, *p* < .001), (Fig. 1A) than RT effect in analysis without the biosignatures, with an adjusted HR = 0.6 (95% CI 0.4–1.0, *p* = .072) (Fig. 1B).

This multivariable analysis was repeated as complementary propensity score adjusted analyses that had consistent results whether the Cox regression analysis was stratified by propensity score ranges or adjusted by propensity score as a covariate. (Supplemental Tables 10, 11). Similar results for the association of the biosignatures with LRR risk were also observed in women aged 50 years and older (Supplemental Fig. 2 and Supplemental Table 12).

### Locoregional recurrence risk—categorical test results

**Fig. 2:**
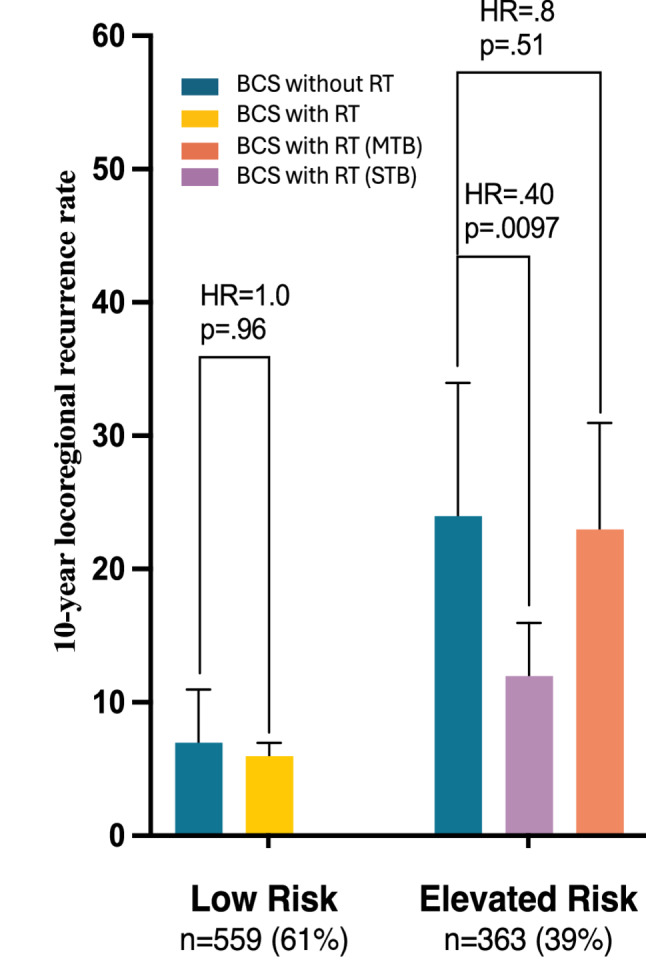
10-year LRR in biosignature risk groups by RT: (1) Low Risk (DS ≤ 5) with RT (yellow) and without RT (blue), (2) Elevated Risk (DS > 5) without RT (blue) with Significant Therapeutic Benefit (STB) (RRI ≤ 5) (periwinkle), 2) Elevated Risk with Minimal Therapeutic Benefit (MTB) (RRI > 5) (orange). There were 186 patients treated without RT, where those with an Elevated Risk (DS > 5) versus a Low Risk (DS ≤ 5) were associated with an increased HR = 4.8 (95% CI 1.8–12.5), *p* = .0014. See supplemental Table 11

To demonstrate the effect of DS and RRI on absolute LRR risk as a secondary and interpretive analysis, the continuous DS and RRI were additionally categorized into risk groups expected to have lower (DS ≤ 5) and higher (DS > 5) LRR risk, and the continuous RRI was categorized into a higher RT effect group (RRI ≤ 5) and a lower RT effect group (RRI > 5). Patients treated without RT in the Elevated Risk (DS > 5) group (*n* = 363, 41%) had an increased LRR risk (HR = 4.8; 95% CI 2.2–10.4; *p* = .0014) compared to the Low Risk (DS ≤ 5) group. The corresponding average 10-year LRR risks without RT were 24% (95% CI 15–34%) in the Elevated Risk group and 7% (95% CI 2–11%) in the Low Risk group, as shown in the bar plot in Fig. 2A and associated Kaplan-Meier plot (Supplemental Fig. 3).

For patients in the categorical Low Risk (DS ≤ 5) group (*n* = 559, 61%), neither RT (HR = 0.8; *p* = .69) nor ET (HR = 0.6; *p* = .33) was associated with a significant reduction in LRR risk in multivariable analysis (Table 3A). The corresponding 10-year LRR risks were 7% without RT and 6% with RT (1% difference, p-log rank=0.96) (Supplemental Table 13).

In contrast, overall patients in the Elevated Risk (DS > 5) group had a significant reduction in LRR risk from RT (HR = 0.5; 95% CI 0.3 to 0.9; *p* = .034) but only trended for a reduction in LRR risk from ET (HR = 0.6; 95% CI 0.4–1.0; *p* = .12) (Table 3B). When the Elevated Risk (DS > 5) group was further categorized into differential RT effect groups, those patients in the higher RT effect group (*n* = 187/290, 64% of DS Elevated Risk group) who were treated with RT had a significant absolute reduction (12%) in LRR risk (HR = 0.4, 95% CI 0.2–0.7) (Fig. 2 and Supplemental Fig. 3). The corresponding average 10-year LRR risks for this group were 24% (95% CI 11–32%) without RT and 12% (95% CI 8–16%) with RT. However, for patients in the lower RT effect group (*n* = 103/290, 36% of DS Elevated Risk group), RT was not associated with a statistically significant reduction in LRR risk (HR = 0.8; 95% CI 0.4–1.4; *p* =.51), where the corresponding 10-year LRR risk remained at 23% (95% CI 15–31%) with RT (Supplemental Table 13). Similar results were obtained for women aged 50 years or older, where there was not a statistically significant difference in LRR risk among patients treated with RT versus without RT among those in the Low Risk group (3% absolute) (Supplemental Tables 13 and Supplemental Fig. 5). Similar results were also obtained for alternative DS = 3.8 and RRI = 3.8 thresholds (Supplemental Fig. 4 and Supplemental Table 14).

## Discussion

For patients with early-stage HR+/HER2- IBC, radiation oncologists routinely address the role of RT after BCS to decrease the risk of breast cancer recurrence in consultation with their patients. Prior studies have shown that adjuvant RT on average reduces the relative risk of LRR by 50% or more, but when the absolute reduction in LRR risk is less than 10%, RT has no impact on survival. When faced with a higher risk of LRR without RT many patients elect RT, although the absolute reduction in LRR from RT may be relatively small as demonstrated in CALGB 9343 [6] and PRIME II [7]. Multiple randomized clinical trials also demonstrated that on average 13% of node-negative patients undergoing BCS had locoregional recurrences prevented by adjuvant RT [1].

These studies highlight the heterogeneity in treatment response and the need for tools that predict recurrence risk and treatment outcomes based on the assessment of individual tumor biology. Several assays are being studied that have been shown to be prognostic but have not shown to be predictive of RT benefit [13–15]. Consistent with this, a recently published MSKCC study demonstrated that genomic low risk (Oncotype DX < 18) [16] patients had a 1.3% per year LRR risk without RT but had a clinically significant reduction in LRR risk to 0.2% per year from RT (*p* < .001) [17]. Thus, there remains a clinical need for validating personalized testing that identifies patients who have a low recurrence risk with no to minimal benefit from RT and patients who have elevated recurrence risk despite standard treatment. This is where a novel approach using a multi-omic assay combining NGS, multiplex proteomics, and spatial biology, has the promise of better-informed shared decision-making by not only providing improved prognostic information but by predicting RT treatment benefit.

In the present analysis, the multi-omic test identified patients with low and elevated LRR risks. Importantly, in patients categorized into the Low Risk (DS ≤ 5) group, RT had no significant reduction in LRR risk after adjusting for clinicopathology and adjuvant therapy (ET and CT), while patients in the Elevated Risk group (DS > 5) had a statistically significant reduction in LRR risk from RT. Thus, the DS biosignature identified low-risk patients in whom no statistically significant difference in LRR risk from RT was observed (absolute difference = 1%, p_logrank_ = 0.96). This is an important finding as previous studies that have attempted to identify patients at low-risk for LRR following BCS have struggled to identify patients that have no to minimal benefit from RT.

Further, the test predicted differential RT effect on LRR risk. The multivariable analysis demonstrated that continuous RRI had a statistically significant interaction with RT, where patients treated with RT who had higher RRI results had increased LRR risk. Among patients in the Elevated Risk group (DS > 5), most patients had a statistically significant reduction in LRR risk from RT; however, no statistically significant reduction in LRR risk from RT was observed in a subset of these patients (RRI > 5), leading to a higher LRR risk remaining after BCT. This is an important finding as the test identifies patients with elevated recurrence risk and had a statistically significant reduction in LRR risk from RT, as well as a subset of patients for whom no statistically significant reduction in LRR risk from RT was observed and may require more aggressive treatment.

In the present study, multivariable analysis demonstrated that the test was prognostic for LRR risk and predictive of RT benefit after adjusting for clinicopathologic factors and adjuvant treatments (ET and CT). Comparison of the multivariable analyses for LRR risk with and without the test demonstrated that the test added statistically significant information to CP factors. Findings were consistent in the subset of women 50 years and older where clinicopathologic factors were not associated with LRR after adjusting for the test. Similar results were obtained using the propensity score adjusted analyses to account for variations in CP factors by treatment, indicating the test provided significant prediction despite associations between CP factors and RT treatment in this non-randomized study.

There are limitations to the present analysis. First, this is a cross-validation study using a retrospective analysis of non-randomized treatment and therefore has intrinsic limitations of such an approach, including changes in treatment over time. Additionally, treatment with RT and endocrine therapy was not rules-based and endocrine therapy compliance was not assessed. As such, differences were noted with respect to clinicopathologic and treatment features between sites and those receiving RT (vs. no RT), reflecting the real-world heterogeneity in clinical decision making. However, the subsequent propensity- adjusted analyses were designed to account for these imbalances and had consistent results, affirming the findings for the biosignatures. Some histologic features, such as tumor focality (unifocal vs. multifocal disease), were not systematically collected across cohorts and therefore could not be evaluated in the current analysis. Moreover, the study endpoint was intentionally limited to invasive LRR (DCIS recurrences were not included), consistent with NCCN treatment goals. Finally, LRR rates seen in this analysis are higher than in more modern trials, reflecting differences in historic treatment practices and advances in treatment. Finally, the selection of categorical thresholds for both DS and RRI within the same dataset used for performance evaluation raises the possibility of optimism bias, even in the context of cross-validation. However, despite these limitations, the results of the present analysis are promising regarding the use of multi-omic biosignatures in the management of patients with early-stage HR+/HER2-negative invasive breast cancer.

## Conclusion

This multi-omic assay, integrating targeted RNA sequencing with multiplex protein biomarker assessment, demonstrated improved prediction of 10-year locoregional recurrence and differential RT effect compared to clinicopathological factors alone in a robust cross-validation analysis of a large non-randomized multi-site cohort of patients with early-stage HR+/HER2– invasive breast cancer. The assay identified a clinically useful subset of patients with low 10-year LRR risk who had no statistically significant reduction in recurrence risk from RT, as well as elevated-risk groups, one with significant reduction in LRR risk from RT and another with no statistically significant reduction in LRR risk from RT. Ongoing studies will provide further clinical validation, and prospective clinical trials are being planned to provide further clinical validation and assessment of clinical utility.

**Table 1:** Summary of clinicopathology factors and treatment by radiotherapy treatment for eligible cohort

Characteristic	No RT	RT	Total	*p*-value
	*n* (%)	*n* (%)	*n* (%)	ChiSq
*Age (Years)*				
< 50	10 (5%)	155 (21%)	165 (18%)	< .001
≥ 50	176 (95%)	581 (79%)	757 (82%)	
*Grade (Nottingham Score)*				
< 8 (low-int)	174 (94%)	658 (89%)	832 (90%)	.13
≥ 8 (high)	12 (6%)	77 (11%)	89 (10%)	
Missing	0 (0%)	1 (0%)	1 (0%)	
*Tumor Size (pT Stage)*				
pT1	161 (87%)	665 (90%)	826 (89%)	.037
pT2	24 (13%)	56 (8%)	80 (9%)	
Missing	1 (1%)	15 (2%)	16 (2%)	
*Nodes (pN Stage)*				
pN0	147 (79%)	592 (80%)	739 (80%)	< .001
pN1	5 (3%)	99 (13%)	104 (11%)	
pNX	34 (18%)	45 (6)	79 (9%)	
*Race*				
White*	160 (86%)	675 (92%)	835 (91%)	.084
Black	13 (7%)	33 (4%)	46 (5%)	
Asian	3 (2%)	7 (1%)	10 (1%)	
Other	8 (4%)	15 (2%)	23 (2%)	
Unknown	2 (1%)	6 (1%)	8 (1%)	
*Radiation therapy*				
No	186 (100%)	0 (0%)	186 (20%)	< .001
Yes	0 (0%)	736 (100%)	736 (80%)	
*Endocrine therapy*				
No	111 (60%)	404 (55%)	515 (56%)	.65
Yes	75 (40%)	332 (45%)	407 (44%)	
*Chemotherapy*				
No	171 (92%)	607 (82%)	778 (84%)	.21
Yes	15 (8%)	129 (18%)	144 (16%)	

Median age 63 years, IQR [53,70], range [25,94]. Median follow-up 10.0 years IQR [5.3, 15.3]

* Directly indicated or assumed based on treating center's regional demographics

**Table 2:** Univariable analysis of clinicopathology risk factors and treatment

	HR (95% CI)	*p*-value
Nottingham Grade (≥ 8 vs. < 8)	1.1 (0.6, 1.8)	.73
Age (per 10 yrs)	0.8 (0.7, 1.0)	.058
Age (< 50 vs. ≥ 50)	1.8 (1.3, 2.5)	.013
Size (T2 vs. T1)	1.1 (0.3, 1.9)	.79
Node Positive (yes vs. no)	0.6 (0.3, 1.1)	.23
RT (yes vs. no)	0.7 (0.5, 1.1)	.15
ET (yes vs. no)	0.7 (0.5, 0.9)	.097
CT (yes vs. no)	1.2 (0.7,1.8)	.59
Events = 91	*n* = 922	

10-year local regional risks as a function of prognosis and RT prediction continuous biosignature scores in women of all ages. There were 922 patients treated with BCS +/-ET, +/- RT, +/- CT, median age (63) IQR (53– 70)

RT radiation therapy, ET endocrine therapy, CT chemotherapy

**Table 3:** Multivariable Cox proportional hazards analysis of clinicopathology factors and treatment for women all ages in low and elevated risk groups

A: Multivariable analysis low risk group			B: Multivariable analysis elevated risk group		
	HR (95%CI)	*p*-value		HR (95%CI)	*p*-value
Nottingham Grade (≤ 8 vs. > 8)	0.4 (0.1, 3.1)	.39	Nottingham Grade (≤ 8 vs. > 8)	1.2 (0.4, 2.1)	.87
Age (< 50 vs. ≥ 50)	2.4 (1.4, 4.1)	.013	Age (< 50 vs. ≥ 50)	1.6 (0.9, 2.8)	.15
Size (T2 vs. T1)	IND (no events)	.99	Size (T2 vs. T1)	1.0 (0.2, 2.1)	.97
Node Positive (yes vs. no)	1.1 (0.2, 5.0)	.85	Node Positive (yes vs. no)	0.7 (0.2, 1.9)	.47
RT (yes vs. no)	0.8 (0.4, 1.8)	.70	RT (yes vs. no)	0.5 (0.3, 0.9)	.034
ET (yes vs. no)	0.6 (0.2, 1.2)	.33	ET (yes vs. no)	0.6 (0.4, 1.0)	.12
*n* = 559	n.events = 38		*n* = 363	n.events = 53	

10-year locoregional risks as a function of prognosis and RT prediction in Low and Elevated Risk groups. There were 922 patients treated with BCS +/, +/- RT, +/- CT, DS Low Risk median age (63) IQR (53 to 70); DS Elevated Risk median age (62) IQR (53–69)

RT radiation therapy, ET endocrine therapy, CT chemotherapy

## Supplementary Information

Below is the link to the electronic supplementary material.

Supplementary Material 1

## Data Availability

The full assay dataset generated and analyzed in this study is not publicly available due to its proprietary nature. De-identified clinical characteristics and outcome data may be shared by the corresponding author upon reasonable request and subject to appropriate data-use agreements. Proprietary assay data, including raw or processed expression outputs, cannot be shared.
