# Supplementary material for: Development and cross-validation of a novel multi-omic assay to assess locoregional recurrence risk and adjuvant therapy benefit in early-stage hormone receptor positive invasive breast cancer patients
Source: Breast Cancer Res. 2026 Feb 18;28:55. doi: 10.1186/s13058-026-02237-4 (PMC12961866; doi:10.1186/s13058-026-02237-4)
Supplement: Supplementary file 1 — Supplementary Material 1 [file 13058_2026_2237_MOESM1_ESM.docx]

***Supplemental Information***

***Study Cohort***

This was a retrospective study on archived tissue samples. The strategy to include multiple sites was chosen to increase patient diversity, patient numbers, and events, including patients treated without RT. A multiple cross-validation approach was used to further improve model generalization to the real-world population. To conserve such resources both whole tissue sections and tissue microarrays were included in the development and cross-validation. Patient diagnosis and treatment were collected along with clinicopathologic data to satisfy patient inclusion and exclusion criteria.

A total of 922 women underwent definitive breast conserving surgery (BCS) for hormone positive (HR+) HER2- invasive breast cancer at four study centers and had formalin fixed paraffin embedded (FFPE) tissue with available treatment and outcome data. Among 922 patients, 835 (91%) self-identified as White or were classified as White. Most patients had T1 (90%) and node-negative (N0 or NX, 91%) disease. High histologic grade (Nottingham grade 8–9) was reported in 89 patients (10%), [**Table 1**](#_Table_1._Summary). All patients were margin negative (no ink on tumor). When patients were stratified by receipt of RT, characteristics of grade, race and ET were similarly distributed between patients treated with and without RT. While 95% (99/104) of patients with positive nodes were treated with RT, 94% (155/165) of patients aged <50 years and 77% of patients aged ≥50 years (581/757) were treated with RT. The median follow-up duration was 10.0 years (IQR, 5.3 to 15.3 years).

Among the 757 patients aged 50 years and older, 338 (45%) received endocrine therapy (ET), 581 (77%) received RT, and 36% (269/757) received both RT and ET. Chemotherapy (CT) was administered to 92 patients (12%), of whom 82 (89%) also received RT. There were 688 (91%) patients who self-identified or were classified as White. Most patients had T1 (91%) and node-negative (N0 or NX, 91%) disease. Higher histologic grade (Nottingham grade 8–9) was reported in 67 patients (9%). When patients were stratified by receipt of RT, characteristics of grade, size, race and ET were similarly distributed between patients treated with and without RT. While 94% (66/70) of patients with positive nodes were treated with RT, 14% (51/354) of patients aged over 50 and under 65 years were treated without RT, compared to 31% (125/403) of patients aged 65 years or older were treated without RT. The median follow-up duration was 10 years (IQR, 5.1 to 13.6 years)**, Supplemental Table 6**.

**Assay Methodology**

***Multiplex Immunofluorescence Assay***

For multiplex Immunofluorescence assay development two intermediate steps namely Chromogenic Immunohistochemistry (IHC) and Singleplex Immunofluorescence were required for robust development of the assay. These steps enabled the identification of antibodies that demonstrated specific and reproducible staining of the target protein markers for the assay. The IHC and singleplex immunofluorescence results were used to determine optimal primary antibody conditions for use in the multiplex immunofluorescence assay. Singleplex IHC assays were developed and for all target biomarkers sensitivity, specificity and accuracy were established. For Immunofluorescence staining the tyramide signal amplification (TSA- based opal method) Opal Polaris 7‐Color Automation IHC Kit; Akoya Biosciences, Marlborough, MA, USA; Catalogue No. NEL871001KT) was used. Staining was performed on the BOND RX (Leica) using custom staining protocols developed by PreludeDx under the manufacture’s guidance. Performance of each antibody was assessed in singleplex IF by comparing it to IHC scores.

Using same Akoya kits multiplex immunofluorescence (MIF) were developed with 5 antibodies and DAPI for nuclear detection for five panels of antibodies. The optimal Opal-antibody pairings were selected based on the anticipated co-expression and abundance of biomarkers in invasive breast cancer tissue and expression presented in IHC and singleplex immunofluorescence. Specifically, biomarkers expected to colocalize within the same cellular compartment were paired with Opals that have distinct spectral properties to prevent overlap. Additionally, low-expressing markers were paired with more intense Opals to enhance spectral acquisition, while high-expressing markers were paired with less intense Opals to balance the overall signal. The Opal fluorophores were used following Akoya's recommendations for use with the Leica BOND RX platform. To evaluate staining performance, the results were compared to the corresponding IF singleplex assay scores. This comparison ensured that the fluorescent markers in each panel accurately reflect the expected expression patterns. To score the digital images generated by the MIF staining and scanning the samples used in the current study scoring algorithms were developed on Akoya InForm image analysis Software. Briefly, the native tissue segmentation and cell segmentation feature of Akoya Inform image analysis Software were utilized to create the stromal and tumor areas and identify individual cells within each region. Scoring thresholds were added under guidance of the pathologists based on the low, moderate and high expression of each protein marker. Additionally, HALO Scoring algorithms were established for clinical workflows and cross-validated to the Inform Scoring using 100 patient samples (Data not shown).

***NGS Targeted library***

Whole RNA sequencing was conducted on RNA extracted from the formalin-fixed paraffin-embedded tissue whole sections. RNA extraction was carried out using the Covaris RNA extraction chemistry and the extracted RNA underwent paired-end sequencing, using the low-input library preparation for the samples using Takera's low-input whole RNA seq library prep kits. Post - sequencing the generated Fastq files underwent the alignment and batch normalization of the data using NFcore pipeline, which was further customized and modified using the computational biology platform PLUTO (https://app.pluto.bio). The aligned data were processed using the SSGSEA and gene set scores for previously identified gene sets^1-3^. Using these gene set scores a refined model was developed as a modified version of the previously published model^1-3^. The refined model had gene sets that had strongest prognostic and predictive value, which were used to design the targeted library levaraging TWIST targeted library chemistry. Samples were re-sequenced using the targeted library at PreludeDx molecular laboratory and the results obtained were compared to the whole RNA sequencing data. The findings demonstrated that the targeted library yielded comparable performance to whole RNA seq, confirming the utility of the targeted library in clinical applications.

***Tissue Sample Acceptance and Sectioning***

To process the samples through RNA seq and MIF, first a tissue section for each sample was stained with H&E. The H&E slide underwent pathology review at PreludeDx to confirm the presence of IBC and to identify and annotate the region of interest (ROI), which included the tumor area and surrounding stromal area. Based on the tumor area and prespecified requirements of the tissue required for adequate RNA extraction, unstained tissue sections were cut for extraction of RNA, and 5 additional sections were cut at a thickness of 5um for MIF. RNA seq was only performed on samples for which the full-face sections were available.

***NGS Sample Processing***

Sections for the RNA seq underwent macrodissection by a trained histotechnologist. RNA extraction was performed using the Covaris R230’s commercially available kit, employing a semi-automated extraction method. The RNA was then prepared for PreludeDx's custom targeted library prep followed by Hybridization.

After enrichment, samples were then prepared for the Sequencer loading pool using a high output kit by Illumina and were sequenced on the NextSeq 550 Dx, following manufacturer’s instructions and protocols. After the sequencing runs were complete, the FASTQ files were processed using the NF-core pipeline and PreludeDx's custom SSGSEA analysis.

***MIF Sample Processing***

Unstained FFPE sections for all the samples from the current study were stained using the custom staining protocols developed by PreludeDx using Akoya's Opal Polaris 7‐Color Automation IHC Kit; Akoya Biosciences, Marlborough, MA, USA; Catalogue No. NEL871001KT following the methods described above using Leica BOND RX. Five (5) unstained sections underwent staining for Five panels each comprised of five (5) antibodies and counterstained with DAPI. Positive and negative controls were included for staining runs. Stained slides were scanned using Phenoimager HT scanner (Akoya). Whole sections and dual core tissue microarrays (TMAs) were both scored using image analysis software with custom scoring algorithms. When utilized, TMAs were constructed of duplicate 1 mm cores per patient which were assayed and then scored.

The protein expression biomarkers were quantitatively assessed in two compartments of the tissue using multiplex immunofluorescence image analysis (digital pathology): 1) Tumor compartment: percentage of tumor cells exhibiting 1+, 2+, or 3+ staining intensity; and 2) Stromal compartment: counts/density of 1+, 2+, or 3+ cells within the defined stromal surface area. These representations were selected based on the expected biological expression pattern and compartmental localization of each biomarker (tumor cell–associated vs stromal/immune cell–associated) and were used as quantitative inputs to generate the assay outputs/biosignature results.

For subjects with more than one tissue sample for a biomarker, the average of the two biomarker scores were used for the final biomarker score. In the case that one of the two scores was missing, the non-missing biomarker score was used. Scans and the scores from controls and sample tissues were reviewed by pathologists and approved. Markers were scored and reported as percentage, intensity, positive or negative or Allred score, depending on the marker by PreludeDx. Consensus scores were used when results from multiple sources existed.

***Biosignature Development***

A multi-omic test was developed and cross-validated in this study. Biomarker data generated by RNA sequencing, multiplex immunofluorescence and spatial biology was combined using the non-linear biologic signature. The biosignature was developed using machine learning techniques with the goal of identifying continuous scores to predict LRR risk and RT response^4-7^ .

A series of literature reviews was conducted to identify molecular markers and clinicopathologic factors associated with locoregional recurrence risk and radiation therapy response for early-stage invasive breast cancer^1-3,8-14^. Selection criteria was based on several factors; (1) potential prognostic utility for identifying invasive breast cancer (IBC) events and interaction with RT; (2) Compatibility of the methodology to assay the biomarkers for clinical workflows; (3) freedom to operate ^1-3,13,14^.

To account for the complex interdependencies of oncogenic signaling pathways, a non-linear modeling framework was employed. The utility of engineered features—comprising individual variables as well as interactions among biomarkers and clinicopathologic factors and treatment was assessed. Unlike traditional linear models that assign independent weights to each biomarker, this approach allows composite feature values to reflect non-linear interactions between sets of genes. Pathways driving LRR prognosis or RT response were constructed from sets of proteins and from GSEA gene sets. Specifically, the model incorporated quantile normalized scores across tissue cohorts for protein expression of genes (Supplemental Table 3), and single sample GSEA (ssGSEA) analysis of gene sets (Supplemental Table 4) that were previously identified using publicly available breast cancer data sets and subsequently validated in independent clinical trial cohorts ^1-3^. The individual GSEA gene sets were combined into two composite scores using pre-defined weightings based on their training and validation in the SweBCG91 randomized clinical trial cohort^1,2^ . In summary, 1,178 patients with lymph-node negative (N0) stage I or IIA breast cancer were randomly assigned between 1991 and 1997 to breast-conserving surgery with or without whole-breast RT and followed for a median time of 15.2 years in the SweBCG91 study. Patients with information of outcome and all included covariates were included (n = 739). Time to locoregional recurrence as the first event within 10 years from the date of diagnosis was used as the primary endpoint for the analysis. The weighted gene sets were associated with likelihood of locoregional recurrence risk and with the likelihood of response from RT to reduce risk of LRR. These gene sets were subsequently combined with spatial proteomic data to ultimately generate the DS and RRI biosignatures and calculate 10-year locoregional recurrence risks after BCS with and without RT. Within the study population, the non-linear biologic signatures were developed with the goal of identifying continuous scores for prognosis and RT response^4-7^. The individual weighted terms from the Cox regression for the prognosis score were negative for decreased LRR risk and positive for increased LRR risk compared to the population baseline in the Cox regression. A nested Cox regression model was used to further evaluate with interactions of individual terms with RT while accounting for the prognosis regression. The individual weighted terms from the Cox regression for the RT response were negative for improved RT response (decreased LRR risk) and positive for resistance to RT response (increased LRR risk) based on the interaction of the RT response terms and RT treatment. In cases of missing data impacting a term in the Cox regression for an individual patient, the individual term for the patient was imputed with a zero to reflect no information other than baseline risk available for that individual sample term.

The Cox regressions were parameterized and tested using multiple cross-validation. The modeling was completed using 500 train-test folds with 70% of the eligible population used for training and 30% used for testing in each cross-validation fold. The consensus result of the biosignature scores were determined as the median of the test result data in cross-validation for each patient.

A continuous score ranging from zero to ten, termed the Decision Score (DS), was reported for each patient as a scaled consensus prognosis result. The threshold between the DS Low and DS Elevated Risk groups was scaled to five (5) with the Low Risk Group including patients with DS ≤5, and the Elevated Risk Group including patients with DS >5. A continuous score ranging from zero to ten, termed the Radio-Resistance Index (RRI), was similarly reported for the response to RT based on the interaction of RT and RRI. The threshold between the low and high RRI groups was scaled to five (5) with the patients with a better RT response RRI ≤5, and a limited therapeutic benefit from RT response RRI >5.

**Biostatistics**

***Endpoints***

The primary end point of this study was invasive locoregional recurrence (LRR) free survival. LRR was histologically defined as confirmed invasive disease in the ipsilateral breast or regional lymph nodes including axillary, supraclavicular, infraclavicular, and internal mammary lymph nodes. Node positivity was defined as histologic confirmation of tumor cells in one or more of the regional lymph nodes. Invasive LRR risk was calculated using survival analysis of all first ipsilateral local or regional IBC events after primary IBC, which were right censored. *In situ* recurrence was not included in the primary end point. Distant metastatic (DM) events included all distant metastatic events after the primary IBC. LRR events that occurred after distant metastatic events were censored. Contralateral invasive breast event risk was also assessed. Analyses were based on time from primary IBC diagnosis to recurrence. If a patient did not have any subsequent event, censoring occurred at death or last follow-up.

***Survival Analysis***

Kaplan-Meier analyses were used to estimate 10-year LRR risks for patient subsets using the R library survival. Log-rank testing was used to assess differences between survival curves for categorical risk and treatment groups. For Kaplan–Meier survival analyses, 95% confidence intervals were obtained using a 1,000-fold stratified nonparametric bootstrap, in which sampling was stratified by study centers, and Bias-corrected and accelerated (BCa) confidence intervals were reported using the R library boot. BCa confidence intervals were reported because they adjust for both bias and skewness in the bootstrap distribution, yielding improved accuracy over standard percentile-based intervals.

Hazard ratios for locoregional recurrence risk and 95% confidence intervals were estimated using Cox proportional hazards regression models in the R library survival. The time origin was defined as the date of surgery. Patients were censored at the time of distant metastasis, or last follow-up or death in the absence of an event. The proportional hazards assumption was evaluated using scaled Schoenfeld residuals calculated using the R library survival. Global Schoenfeld residual estimates were used to test the proportional hazards assumption. All models satisfied the assumption (global p > 0.05). Ninety-five percent (95%) confidence intervals for Cox proportional hazards were calculated using a 1,000-fold stratified nonparametric bootstrap, in which sampling was stratified by study centers, and BCa interval reporting using the R library boot.

The Cox proportional hazards models with clinicopathology and treatment versus clinicopathology, treatment, and biosignatures were compared to determine whether adding biological signatures improved prediction of locoregional recurrence over conventional factors. The models were considered nested as the first model included clinicopathologic variables and treatment factors (“base model”) and the second model added the biosignatures to the same variables (“expanded biosignature model”) so that the base model is entirely included within the expanded model, differing only by the addition of the biosignature terms. The nested models were compared using a likelihood ratio test to assess whether the expanded model provided a statistically significant improvement in model fit compared with the base model. A significant result for the likelihood ratio test indicated that inclusion of the biosignatures DS and the interaction with DS and RT added meaningful prognostic and predictive information beyond standard clinicopathologic features and treatment.

***Propensity Score Analysis***

As the analysis was completed in a non-randomized observational study, patients who received radiotherapy (RT) had treatment-selection bias, where those treated with RT differed systematically from those who did not (see Table 1). This treatment-selection bias could impact observed differences locoregional recurrence, as they may be due to RT or due to underlying differences between patients. Propensity score adjustment using quintile stratification was used to help control for differences in baseline characteristics associated with RT treatment. The propensity score stratification grouped patients into strata according to how likely they were to receive RT. By comparing patients within strata of similar likelihood of receiving RT, this method reduced confounding and allowed more reliable estimation of the association between RT and LRR in a non-randomized study. Specifically, the propensity score was calculated using a logistic regression model for RT (yes/no) that was fit using generalized linear model (GLM) with clinicopathologic factor and treatment, using the R library stats (**Supplemental Table 9**). The resulting propensity score was incorporated in multivariable Cox proportional hazards analysis of locoregional risk stratified by quintile of propensity score. For completeness, the propensity score was also included as a continuous covariate.

**SUPPLEMENTAL RESULTS**

***Locoregional Recurrence in women aged 50 years and older***

In line with the results observed across all ages, the association between the biosignature and LRR risk was also consistently demonstrated in women aged 50 years and older, where increasing continuous DS was associated with higher LRR risk (adjusted HR=3.7 per 5 units; 95% CI, 2.3 to 6.2; p<.001) in a multivariable analysis (**Supplemental Figure 2A**, **Supplemental Table 12**). RT was associated with a lower LRR risk (adjusted HR=0.2; 95% CI, 0.1 to 0.4; p<.001); however, the continuous RRI had a significant interaction with RT (p_int_<.001), where among patients treated with RT, increasing RRI was associated with higher LRR risk (HR=4.0 per 5 units; 95% CI, 1.6 to 8.6). Endocrine therapy was not associated with a significantly lower LRR risk (adjusted HR=0.6; 95% CI, 0.4 to .9; p=.11). None of the clinicopathological factors were associated with LRR risk in univariable analysis. However, RT was associated with lower LRR risk (HR= 0.6, 95% CI, 0.4 to 0.9; p=0.037) (Supplemental Table 8). The other clinicopathology risk factors were also not significantly associated with LRR risk. (**Supplemental Table 12**).

***Alternative* Risk Groups for Continuous RRI and DS**

When continuous RRI was further categorized with alternative thresholds, LRR risk after RT varied with RRI. For patients in the Elevated Risk (DS>5) group, the 10-year LRR risk after RT increased from 13% for RRI≤3.8 to 19% for RRI >3.8 ([Supplemental](#_Supplemental_Table_XX) Table 14). For patients with DS ≤5, neither RT (HR=0.9, p=0.73) nor ET (HR=0.6, p=.24) were associated with a statistically significant reduction in LRR risk for patients in the low risk DS group (DS ≤3.8) in the eligible cohort (Supplemental [Figure 4](#_Supplemental_Figure_3:)) . Similar results were obtained when continuous DS was assessed with an alternative threshold (DS=3.8) for categorial DS risk groups..

### **Supplemental Table 1.** Clinicopathology and treatment characteristics by study centers for eligible cohort.

|  | **NYU** | **Sweden** | **BAL** | **USF** | **TOTAL** | **Chi Square** |
| --- | --- | --- | --- | --- | --- | --- |
| **Characteristic** | **n (%)** | **n (%)** | **n (%)** | **n (%)** | **n (%)** | **p-value** |
| **Age (Years)** | | | | | | |
| **<50** | 7 (10%) | 115 (21%) | 17 (19%) | 26 (12%) | 165 (18%) | <.001 |
| **≥50** | 60 (90%) | 439 (79%) | 72 (81%) | 186 (88%) | 757 (82%) |  |
| **Tumor Grade (Nottingham Score%)** | | | | | | |
| **<7** | 54 (81%) | 448 (81%) | 51 (57%) | 142 (67%) | 695 (75%) | <.001 |
| **≥7** | 13 (19%) | 105 (19%) | 38 (43%) | 70 (33%) | 226 (25%) |  |
| **Tumor Grade (Nottingham Score%)** | | | | | | |
| **<8** | 64 (96%) | 516 (93%) | 71 (80%) | 181 (85%) | 823 (90%) | <.001 |
| **≥8** | 3 (4%) | 37 (7%) | 18 (20%) | 31 (15%) | 89 (10%) |  |
| **Tumor Size (pT Stage%)** | | | | | | |
| **pT1** | 56 (84%) | 531 (96%) | 67 (75%) | 172 (81%) | 826 (90%) | <.001 |
| **pT2** | 11 (16%) | 9 (2%) | 21 (24%) | 39 (18%) | 80 (9%) |  |
| **Missing** | 0 (0%) | 14 (3%) | 1 (1%) | 1 (0%) | 16 (2%) |  |
| **Lymph Nodes (pN Stage%)** | | | | | | |
| **pN0** | 52 (78%) | 427 (77%) | 65 (73%) | 195 (92%) | 739 (80%) | <.001 |
| **pN1** | 5 (7%) | 71 (13%) | 17 (19%) | 11 (5%) | 104 (11%) |  |
| **pNX** | 10 (15%) | 56 (10%) | 7 (8%) | 6 (3%) | 79 (9%) |  |
| **Race** | | | | | | |
| **White*** | 52 (78%) | 554 (100%) | 71 (80%) | 158 (75%) | 835 (91%) | <.001 |
| **Black** | 6 (9%) | 0 (0%) | 16 (18%) | 24 (11%) | 46 (5%) |  |
| **Asian** | 2 (3%) | 0 (0%) | 0 (0%) | 8 (4%) | 10 (1%) |  |
| **Other** | 7 (10%) | 0 (0%) | 0 (0%) | 16 (8%) | 23 (2%) |  |
| **Unknown** | 0 (0%) | 0 (0%) | 2 (2%) | 6 (3%) | 8 (1%) |  |
| **Radiation Therapy** | | | | | | |
| **No** | 41 (61%) | 83 (15%) | 33 (37%) | 29 (14%) | 186 (20%) | <.001 |
| **Yes** | 26 (39%) | 471 (85%) | 56 (63%) | 183 (86%) | 736 (80%) |  |
| **Endocrine Therapy** | | | | | | |
| **No** | 41 (61%) | 393 (71%) | 24 (27%) | 57 (27%) | 515 (56%) | <.001 |
| **Yes** | 26 (39%) | 161 (29%) | 65 (73%) | 155 (73%) | 407 (44%) |  |
| **Chemotherapy** | | | | | | |
| **No** | 60 (90%) | 516 (93%) | 55 (62%) | 147 (69%) | 778 (84%) | <.001 |
| **Yes** | 7 (10%) | 38 (7%) | 34 (38%) | 65 (31%) | 144 (16%) |  |

### **Supplemental Table 2.** Signaling axes.

| **Signaling Axes** | **Dysregulated Pathways Captured by the Biosignature** |
| --- | --- |
| **1. Cell-Cycle Control/Proliferation** | |
|  | G1/S transition axis |
|  | G₂/M Checkpoint axis |
|  | C-myc Transcriptional Amplification Axis |
|  | DNA Replication Axis |
|  | Oncogenic Stress Response Axis |
| **2. Metabolic Reprogramming** | |
|  | Aerobic Glycolysis |
|  | Hypoxia Response |
|  | Oxidative Phosphorylation |
|  | ALDHA1 Driven stem cell phenotype and retinoic acid synthesis |
| **3. Stress Response Pathways and Cell Survival** | |
|  | Oxidative Stress Response |
|  | DNA Damage Response |
|  | Senescence and senescence-associated secretory phenotype |
|  | Hypoxia Response |
|  | JAK- STAT pathway |
|  | MAPK pathway - Families of pathway here are - HER2 KRAS, ASK1/JNK activation, p38/JNK activation, RAS/RAF/MEK/ERK pathway |
|  | NF-kb signaling |
|  | PI3K → AKT → mTOR pathway |
| **4. Immune Regulation/Immune-Tumor Cell Interaction/Crosstalk** | |
|  | Type I Interferon Response |
|  | Type II Interferon Response |
|  | IL-2 / STAT5 Signaling |
|  | Cytokine receptor interaction |
|  | PD- Checkpoint signaling |
|  | T- cell exhaustion |

Supplemental Table 3: List of the 17 protein biomarkers analyzed for the biosignature.

NOTE: PanCK is included as a control

| **Target** | **Full name** |
| --- | --- |
| CD4 | Cluster of Differentiation 4 |
| CD8 | Cluster of Differentiation 8 |
| FOXP3 | Forkhead box P3 |
| PDL1 | Programmed cell death 1 ligand |
| PD1 | Programmed cell death 1 |
| ER | Estrogen receptor |
| PR | Progesterone Receptor |
| ALDH1A1 | Aldehyde dehydrogenase 1 family member A1 |
| IRF-9 | Interferon regulatory factor 9 |
| Ki-67 | marker of proliferation Ki-67 |
| FOXA1 | Forkhead box A1; hepatocyte nuclear factor 3 alpha |
| COX2 | Cyclooxygenase-2 |
| GLUT-1 | glucose transporter-1 |
| HER2 | Human Epidermal Growth Factor Receptor-2 |
| P16INK4a | cyclin-dependent kinase inhibitor 2A |
| SIAH2 | seven in absentia homolog 2 |
| PanCK | Pan cytokeratin |

**Supplemental Table 4: List of genes for which mRNA is measured using Next Generation Sequencing for biosignature**

| **GENES** | | | | | | | | | | |
| --- | --- | --- | --- | --- | --- | --- | --- | --- | --- | --- |
| ZNF395 | CAPN2 | CLEC4E | E2F5 | GPC1 | ITGB7 | MAOA | PCSK5 | RAI2 | SLC26A3 | TNFRSF17 |
| AASS | CARD11 | CLIP4 | ECHDC2 | GPD1 | ITIH5 | MAP3K12 | PDCD4 | RASGRP1 | SLC39A8 | TNFRSF1A |
| ABCA8 | CASP3 | COBLL1 | ELOVL2 | GSTP1 | ITPKB | MCM4 | PDE3B | RBP1 | SLC4A7 | TNFSF13 |
| ACTL6A | CBX6 | COL14A1 | ENPP2 | GZMB | JAK2 | ME1 | PDGFC | RBP4 | SMO | TNN |
| ACTN1 | CCL5 | COPB2 | EOMES | GZMK | JCHAIN | MFAP4 | PDGFD | REL | SMOC2 | TOB1 |
| ADH1B | CCNB2 | CP | ERGIC1 | H2BC10 | KCNA3 | MMP1 | PDIA4 | RGS10 | SP140 | TOX |
| ADIRF | CCR7 | CPA3 | ESM1 | H2BC5 | KCNK1 | MMP13 | PELP1 | RHOBTB3 | SPP1 | TP53BP1 |
| AFF3 | CCT2 | CRIP1 | ESR1 | HIGD1A | KCNN4 | MMP7 | PGR | RIPK3 | SRF | TPBG |
| AGR2 | CD2 | CSNK1G3 | EVL | HIP1 | KDM5B | MOCOS | PHLDA1 | RPL13 | SRI | TPX2 |
| AHNAK2 | CD24 | CTTN | EXPH5 | HMGB1 | KIF13B | MPC2 | PHTF2 | RPL13A | SRM | TSPAN13 |
| AP1M2 | CD274 | CX3CR1 | FABP4 | HMGB3 | KIF15 | MRPS21 | PIM2 | RPS2 | STAM2 | TSPAN6 |
| APMAP | CD28 | CXCL10 | FAM162A | HMGCS2 | KIF16B | MT1E | PLCE1 | RPS3 | STAT5B | TXNDC16 |
| AQP3 | CD38 | CXCL13 | FAM171A1 | HOXB2 | KIF22 | MUC1 | PLK2 | RTCB | STAT5B | TXNDC5 |
| AREG | CD3E | CXCL14 | FAM30A | ID2 | KLF5 | MYB | POC1B | RTN1 | STC2 | UAP1 |
| ATG5 | CD3G | CXCL9 | FBLN1 | IDO1 | KLF9 | MZB1 | PON2 | RUVBL1 | STK11 | UBD |
| ATP1A2 | CD86 | CXCR6 | FCGR1A | IER3 | KLHDC7B | NAT1 | PPA1 | S100P | STK3 | UBE2C |
| ATP1B1 | CD8A | CYBRD1 | FECH | IFI27 | KLRC2 | NBN | PPARG | SCARA3 | SVEP1 | UBE2C |
| ATRNL1 | CDC42BPA | CYFIP2 | FGR | IFT46 | LAMA2 | NFATC1 | PPP1R16B | SCD | SYNC | UCK2 |
| AURKA | CDCA7 | CYP4B1 | FHL1 | IFT46 | LAMP5 | NME5 | PRDX4 | SCRN1 | SYTL4 | USP13 |
| BBS1 | CDH1 | DHRS2 | FOS | IGF1 | LARGE1 | NOTCH1 | PROM1 | SCUBE2 | TAT | USP34 |
| BCL11A | CDH2 | DHRS7 | FRY | IGF1 | LFNG | NRCAM | PRSS23 | SEL1L3 | TBL1X | UST |
| BIRC3 | CEACAM6 | DIXDC1 | FYN | IKZF3 | LILRB4 | NUP107 | PTGER3 | SELL | TENT5C | WDR19 |
| BMERB1 | CENPF | DLG5 | G0S2 | IL27RA | LIMCH1 | NYNRIN | PTHLH | SERPINA1 | TFF3 | WNT2 |
| BTN2A2 | CFAP69 | DNAJC12 | GATA1 | IL2RA | LIN9 | OAT | PTN | SESN1 | TLX1 | WNT5A |
| BTN3A1 | CHPT1 | DNAJC15 | GATA3 | IL6ST | LMO4 | OGN | PTPN2 | SFRP4 | TM4SF1 | XCL1 |
| C1orf21 | CIDEC | DOCK2 | GATM | IMPDH2 | LRRC17 | OMD | PTPRC | SH3BP4 | TMC5 | YBX3 |
| C3orf14 | CILP | DPT | GDE1 | IPO9 | LRRC32 | OSER1 | PTTG1 | SH3BP5 | TMEM100 | ZAP70 |
| CADM1 | CIRBP | DTNA | GFRA1 | IRF4 | LTBP3 | PALLD | PXDN | SHH | TMEM156 | ZEB2 |
| CAMK2N1 | CLC | DUSP5 | GLDC | IRF8 | LYZ | PALS2 | PYCARD | SHROOM3 | TMF1 | ZHX2 |
| CAP2 | CLEC12A | DZANK1 | GLI3 | ITGAL | MANSC1 | PARP1 | PYGL | SLC1A4 | TMX1 |  |

### **Supplemental Table 5.** Ipsilateral breast, contralateral breast and distant metastatic events within 10 -years.

**HR+/HER2–, BCS, all ages, events within 10-years**

| Events within 10-years | No RT | RT | Total |
| --- | --- | --- | --- |
|  | n=186 | n=736 | n=922 |
| Ipsilateral Local IBC | 19  (10%) | 54  (7%) | 73 (8%) |
| Ipsilateral Regional IBC | 0 [3] (0%) [2%] | 1 [5]  (<1%) [<1%] | 1 [8]  (<1%) [<1%] |
| Contralateral IBC | 10 (5%) | 33  (4%) | 43  (5%) |
| Distant Metastasis | 7 (4%) | 29  (4%) | 36  (4%) |

### Number of first events within 10 years and raw percentage (%), number of censored events [nn] and raw percentage [%].

| 10-year Risks | No RT | RT | No ET | ET |
| --- | --- | --- | --- | --- |
|  | % 10-year risk  (95%CI) | % 10-year risk  (95%CI) | % 10-year risk  (95%CI) | % 10-year risk  (95%CI) |
|  | n=186 | n=736 | n=515 | n=407 |
| Ipsilateral Locoregional IBC | 13%  (9%, 18%) | 9%  (8%,11%) | 11%  (9%, 14%) | 8%  (6%, 11%) |
| Contralateral IBC | 7%  (4%, 11%) | 6%  (4%, 7%) | 7%  (5%, 9%) | 4%  (2%, 6%) |
| Distant Metastasis | 4%  (2%, 7%) | 5%  (4%, 6%) | 5%  (3%, 6%) | 5%  (3%, 7%) |

### 10 year risks and 95% confidence intervals by event type

### **Supplemental Table 6.** Summary of clinicopathology factors and treatment for women age ≥50 years by radiotherapy treatment.

|  | **No RT** | **RT** | **Total** | **Chi Square** |
| --- | --- | --- | --- | --- |
|  | **n (%)** | **n (%)** | **n (%)** | **p-value** |
| **Age (Years)** | | | | |
| **<65** | 51 (29%) | 303 (52%) | 354 (47%) | <.001 |
| **≥65** | 125 (71%) | 278 (48%) | 403 (53%) |  |
| **Tumor Grade (Nottingham Score)** | | | | |
| **<7 (low-int)** | 141 (80%) | 437 (75%) | 578 (76%) | 0.20 |
| **≥7 (high)** | 35 (20%) | 144 (25%) | 179 (24%) |  |
| **Tumor Grade (Nottingham Score)** | | | | |
| **<8 (low-int)** | 166 (94%) | 524 (90%) | 690 (91%) | .12 |
| **≥8 (high)** | 10 (6%) | 57 (10%) | 67 (9%) |  |
| **Tumor Size (pT Stage)** | | | | |
| **T1** | 155 (88%) | 534 (92%) | 689 (91%) | .08 |
| **T2** | 20 (11%) | 40 (7%) | 60 (8%) |  |
| **missing** | 1 (1%) | 7 (1%) | 8 (1%) |  |
| **Lymph Nodes (pN Stage)** | | | | |
| **pN0** | 139 (79%) | 476 (82%) | 615 (81%) | .0017 |
| **pN1** | 4 (2%) | 66 (11%) | 70 (9%) |  |
| **pNX** | 33 (19%) | 39 (7%) | 72 (10%) |  |
| **Race** | | | | |
| **White*** | 154 (88%) | 534 (92%) | 688 (91%) | .28 |
| **Black** | 11 (6%) | 26 (4%) | 37 (5%) |  |
| **Asian** | 2 (1%) | 4 (1%) | 6 (1%) |  |
| **Other** | 7 (4%) | 13 (2%) | 20 (3%) |  |
| **Unknown** | 2 (1%) | 4 (1%) | 6 (1%) |  |
| **Radiation Therapy** | | | | |
| **No** | 176 (100%) | 0 (0%) | 176 (23%) | <.001 |
| **Yes** | 0 (0%) | 581 (100%) | 581 (77%) |  |
| **Endocrine Therapy** | | | | |
| **No** | 107 (61%) | 312 (54%) | 419 (55%) | .12 |
| **Yes** | 69 (39%) | 269 (46%) | 338 (45%) |  |
| **Chemotherapy** | | | | |
| **No** | 166 (94%) | 499 (86%) | 665 (88%) | .0041 |
| **Yes** | 10 (6%) | 82 (14%) | 92 (12%) |  |

Median age 65 years IQR [59,72], range [50,94]. Median follow-up 10.0 years IQR [5.1,13.6]

### **Supplemental Table 7.** Ipsilateral breast, contralateral breast and distant metastatic events within 10 -years for Women 50 years and older

| Events within 10-years | No RT | RT | Total |
| --- | --- | --- | --- |
|  | n=176 | n=581 | n=757 |
| Ipsilateral Local IBC | 18  (10%) | 35  (6%) | 53  (7%) |
| Ipsilateral Regional IBC | 0 [3] (0%) [2%] | 1 [3]  (<1%) [1%] | 1 [6]  (<1%) [1%] |
| Contralateral IBC | 10 (6%) | 31  (5%) | 41  (5%) |
| Distant Metastasis | 7 (4%) | 21  (4%) | 28  (4%) |

### Number of first events within 10 years and raw percentage (%), number of censored events [nn] and raw percentage [%].

| 10-year Risks | No RT | RT | No ET | ET |
| --- | --- | --- | --- | --- |
|  | % 10-year risk  (95%CI) | % 10-year risk  (95%CI) | % 10-year risk  (95%CI) | % 10-year risk  (95%CI) |
|  | n=176 | n=581 | n=419 | n=338 |
| Ipsilateral Local IBC | 13%  (9%, 18%) | 8%  (6%,10%) | 11%  (8%, 13%) | 7%  (4%, 10%) |
| Contralateral IBC | 8%  (4%, 12%) | 7%  (5%, 9%) | 8%  (6%, 11%) | 5%  (3%, 7%) |
| Distant Metastasis | 5%  (2%, 7%) | 5%  (3%, 6%) | 4%  (3%, 6%) | 5%  (3%, 7%) |

### 10 year risks and 95% confidence intervals by event type

**Supplemental Table 8. Univariable Analysis for Age≥50 years**

|  | **HR  (95% CI)** | **p-value** |
| --- | --- | --- |
| Nottingham Grade  (≥7 vs <7) | 1.3  (0.8, 2.0) | .32 |
| Nottingham Grade  (≥8 vs <8) | 1.6  (0.6, 2.6) | .25 |
| Age  (per 10 yrs) | 1.1  (0.8, 1.4) | .54 |
| Size  (T2 vs T1) | 1.3  (0.0, 2.3) | .61 |
| Node Positive (yes vs no) | 0.4  (0.1, 0.9) | .15 |
| RT  (yes vs no) | 0.6  (0.4, 0.9) | .037 |
| ET  (yes vs no) | 0.6  (0.4, 0.9) | .10 |
| CT  (yes vs no) | 1.4  (0.6, 2.3) | .43 |
| events=63 | n=757 |  |

### **Supplemental Table 9.** Multivariable Cox proportional hazards analysis of LRR for clinicopathology factors and treatment alone or clinicopathology factors with biosignature for all ages.

**Update to Supplemental Table 9.**

| **A: Multivariable analysis without biosignatures** | | |  | **B: Multivariable analysis with biosignatures continuous** | | | |
| --- | --- | --- | --- | --- | --- | --- | --- |
|  | HR  (95%CI) | p-value |  | |  | HR  (95%CI) | p-value |
| Nottingham Grade  (8-9 vs <8) | 1.1  (0.5, 1.8) | .87 |  | | Nottingham Grade  (8-9 vs <8) | 0.8  (0.3, 1.5) | .55 |
| Age  (<50 vs ≥50 yrs) | 1.8  (1.1, 2.6) | .015 |  | | Age  (<50 vs ≥50 yrs) | 2.0  (1.4, 3.0) | .0042 |
| Tumor Size  (T2 vs T1) | 1.0  (0.3, 1.9) | .94 |  | | Tumor Size  (T2 vs T1) | 0.9  (0.2, 1.8) | .83 |
| Node Positive (yes vs no) | 0.7  (0.3, 1.3) | .34 |  | | Node Positive (yes vs no) | 0.7  (0.3, 1.4) | .34 |
| RT  (yes vs no) | 0.6  (0.4, 1.0) | .072 |  | | RT  (yes vs no) | 0.2  (0.1, 0.4) | <.001 |
| ET  (yes vs no) | 0.7  (0.5, 1.0) | .19 |  | | ET  (yes vs no) | 0.7  (0.5, 1.0) | .14 |
| events=91 | n=922 |  |  | | DS per 5 units | 3.4  (2.1, 4.9) | <.001 |
|  |  |  |  | | RRI Interaction with RT per 5 units | 3.1  (1.5, 5.7) | .002 |
|  |  |  |  | | events=91 | n=922 |  |
| Multivariable analysis of locoregional (LRR) risks as a function of CP alone and CP with biosignature scores. There were 922 patients treated with BCS + ET, +/- RT, +/- CT, median age (63) range (25 to 94). Likelihood ratio test indicated that the biosignature added not available from CP factors and treatment alone (p<.001, Chi-Squared=34.1, df=2).  A*bbreviations: RT – radiation therapy, ET – endocrine therapy, CT – chemotherapy, df – degrees of freedom.* | | | | | | | |

### **Supplemental Table 10.** Propensity Score adjusted multivariable Cox proportional hazards analysis for LRR with clinicopathology factors and treatment for women of all ages

| **A: Multivariable analysis with biosignatures (continuous) stratified by propensity score** | | |  | **B: Multivariable analysis with biosignatures (continuous) adjusted by propensity score** | | | |  |
| --- | --- | --- | --- | --- | --- | --- | --- | --- |
|  | HR  (95%CI) | p-value |  | | GLM term | Coefficient  (SE) | p-value |  |
| Nottingham Grade  (7-8 vs <8) | 0.8  (0.4, 1.6) | .62 |  | | Nottingham Grade  (7-8 vs <8) | 0.8  (0.3, 1.5) | .61 |  |
| Age  (<50 vs ≥50 yrs) | 2.2  (0.9, 4.7) | .068 |  | | Age  (<50 vs ≥50 yrs) | 2.2  (1.4, 3.5) | .0050 |  |
| Tumor Size  (T2 vs T1) | 0.7  (0.2, 1.8) | .60 |  | | Tumor Size  (T2 vs T1) | 0.9  (0.2, 1.8) | .78 |  |
| Node Positive (yes vs no) | 0.9  (0.3, 2.0) | .82 |  | | Node Positive (yes vs no) | 0.7  (0.3, 1.5) | 0.44 |  |
| RT  (yes vs no) | 0.2  (0.1, 0.4) | <.001 |  | | RT  (yes vs no) | 0.3  (0.1, 0.5) | .0019 |  |
| ET  (yes vs no) | 0.6  (0.4, 1.0) | .11 |  | | ET  (yes vs no) | 0.7  (0.5, 1.0) | 0.18 |  |
| DS per 5 units | 3.2  (2.1, 4.9) | <.001 |  | | DS per 5 units | 3.3  (2.1, 5.0) | <.001 |  |
| RRI Interaction with RT per 5 units | 2.8  (1.3, 5.2) | .0066 |  | | RRI Interaction with RT per 5 units | 3.1  (1.5, 5.8) | .0022 |  |
| events=91 | n=922 |  |  | | Propensity Score | 0.6  (0.1, 3.6) | .54 |  |
|  |  |  |  | | events=91 | n=922 |  |  |
|  |  |  |  | |  |  |  |  |
| **A.** Multivariable analysis for LRR was stratified by quintile of Propensity Score.  **B.** Multivariable analysis for LRR was adjusted for Propensity Score. (See Propensity Score Model in Supplemental Table 9)  **A*bbreviations****: BCS – Breast Conserving Surgery, RT – radiation therapy, ET – endocrine therapy, CT – chemotherapy, LRR – Local Regional Risk, GLM – Generalized Linear Model, PS – Propensity Score,* | | | | | | | | |

### **Supplemental Table 11.** Propensity Score model for RT for women of all ages

| GLM term | Coefficient  (SE) | p-value |
| --- | --- | --- |
| Intercept | -7.5  (3.8) | .048 |
| Age  (qubic spline df=2): term 1 | 13.9  (6.7) | .037 |
| Age  (qubic spline df=2): term 2 | -2.2  (1.2) | .062 |
| Age (<50 vs ≥50 yrs) | 1.0  (.58) | .074 |
| Grade  (qubic spline, df=2): term 1 | 2.3 (2.0) | .25 |
| Grade  (qubic spline, df=2): term 2 | 0.8  (0.5) | .072 |
| Tumor Size  (T2 vs T1) | -1.2 (0.3) | <.001 |
| Node Positive (yes vs no) | 1.7  (0.6) | .0017 |
| ET  (yes vs no) | 0.1  (0.2) | .60 |
| CT  (yes vs no) | 0.3  (0.4) | .46 |
|  | n=922 |  |

| Propensity Score was calculated using a logistic regression model for RT (yes/no) that was fit using generalized linear model (GLM) in R with clinicopathologic factor and treatment.  **A*bbreviations****: BCS – Breast Conserving Surgery, RT – radiation therapy, ET – endocrine therapy, CT – chemotherapy, LRR – Local Regional Risk, GLM – Generalized Linear Model, PS – Propensity Score, df – degrees of freedom.* |
| --- |

### **Supplemental Table 12.** Multivariable Cox proportional hazards analysis of clinicopathology factors and treatment with and without biosignatures for women age≥50 years.

| **A: Multivariable analysis without biosignatures** | | |  | **B: Multivariable analysis with biosignatures continuous** | | | |  |
| --- | --- | --- | --- | --- | --- | --- | --- | --- |
|  | HR  (95%CI) | p-value |  | |  | HR  (95%CI) | p-value | |
| Nottingham Grade (≥8 vs <8) | 1.7  (0.7, 3.1) | .21 |  | | Nottingham Grade | 1.3  (0.6, 2.6) | .55 | |
| Age  (per 10 yrs) | 1.0  (0.8, 1.3) | .98 |  | | Age  (per 10 yrs) | 1.1  (0.8, 1.4) | .58 | |
| Size  (T2 vs T1) | 1.1  (0.1, 2.2) | .85 |  | | Size  (T2 vs T1) | 0.8  (0.2, 1.7) | .73 | |
| Node Positive (yes vs no) | 0.5  (0.1, 1.5) | .23 |  | | Node Positive (yes vs no) | 0.5  (0.1, 1.6) | .24 | |
| RT  (yes vs no) | 0.6  (0.4,0.9) | .054 |  | | RT  (yes vs no) | 0.2  (0.1, 0.4) | <.001 | |
| ET  (yes vs no) | 0.6  (0.4, 1.0) | .14 |  | | ET  (yes vs no) | 0.6  (0.4, 0.9) | .11 | |
| events=63 | n=757 |  |  | | DS per 5 units | 3.7  (2.3, 6.2) | <.001 | |
|  |  |  |  | | RRI Interaction with RT per 5 units | 4.0  (1.6, 8.6) | .0028 | |
|  |  |  |  | | events=63 | n=757 |  | |
| 10-year local regional risks as a function of prognosis and RT prediction continuous biosignature scores in women 50 years of age and older. There were 757 patients treated with BCS +/, +/- RT, +/- CT, median age (65) range (50 to 94). Likelihood ratio test indicated that the biosignature added not available from CP factors and treatment alone (p<.001, Chi-Squared=21.5, df=2).  *Abbreviations: RT – radiation therapy, ET – endocrine therapy, CT – chemotherapy, df – degrees of freedom.* | | | | | | | |  |

### **Supplemental Table 13.** Ten-year locoregional (LRR) risk by categorical biosignature risk groups.

**A. Women of all ages**

| **Thresholds** | | **No RT  LRR % (95%CI) n** | **RT  LRR % (95%CI) n** | **Absolute Differences** | **p-value (log-rank)** | **RT  HR  (95%CI)** | **p-value (wald test)** |
| --- | --- | --- | --- | --- | --- | --- | --- |
| Low Risk Group (DS≤5) |  | 7% (2%, 11%)  113 | 6% (4%, 7%)  446 | 1% | .96 | 1  (0.5, 2.1) | .96 |
| Elevated Risk Group (DS>5) | RRI≤5 | 24% (15%, 34%)  73 | 12% (8%, 16%)  187 | 12% | .012 | 0.4  (0.2, 0.7) | .0097 |
|  | RRI>5 |  | 23% (15%, 31%)  103 | 1% | .51 | 0.8  (0.5, 1.4) | .51 |

**B. Women 50 years and older**

| **Thresholds** | | **No RT  LRR % (95%CI) n** | **RT  LRR % (95%CI) n** | **Absolute Differences** | **p-value (log-rank)** | **RT  HR  (95%CI)** | **p-value (wald test)** |
| --- | --- | --- | --- | --- | --- | --- | --- |
| Low Risk Group (DS≤5) |  | 7%  (3%, 11%) 108 | 4%  (2%, 6%) 347 | 3% | .41 | 0.7 (0.3, 1.4) | .40 |
| Elevated Risk Group (DS>5) | RRI≤5 | 24%  (15%, 34%) 68 | 9%  (5%, 14%) 149 | 15% | .0032 | 0.3 (0.1, 0.5) | .0029 |
|  | RRI>5 |  | 23%  (14%, 32%) 85 | 1% | 0.49 | 0.8  (0.4, 1.4) | .49 |

### Ten-year locoregional (LRR) risk and differences. Log rank test for differences between LRR survival curve by RT. Univariable hazard ratios for RT with 95%CI and p-value within risk groups. In eligible patients treated without RT, those in the DS Elevated Risk group had increased LRR risk compared to those in the DS Low Risk group (HR=4.8; 95%CI 1.8,12.5, p=.0014). there were similar results for women 50 years and older (HR=4.6; 95%CI 1.8,12.1, p=.002).

### *Abbreviations: RT – radiation therapy, ET – endocrine therapy, CT – chemotherapy, DS- Decision Score, RRI- Radiation Resistance Index*

### **Supplemental Table 14.** Ten-year locoregional (LRR) risk with alternative thresholds for categorical biosignature risk groups.

|  | No RT,  10-year LRR risk  (95%CI) | n | RT,  10-year LRR risk  (95%CI) | n |
| --- | --- | --- | --- | --- |
| DS≤3.8 | 7%  (2%, 11%) | 107 | 5%  (4%, 7%) | 423 |
| DS≤5 | 7%  (2%, 11%) | 113 | 6%  (4%, 8%) | 446 |
| DS>5,  RRI≤3.8 | 24%  (15%, 34%) | 73 | 13%  (9%, 18%) | 152 |
| DS>5,  RRI>3.8 |  |  | 19%  (13%, 26%) | 138 |
| DS>3.8,  RRI≤5 | 22%  (14%, 30%) | 79 | 12%  (8%,16%) | 187 |
| DS>3.8,  RRI>5 |  |  | 23%  (15%, 31%) | 103 |

###

### **Supplemental Figure 1.** Remark diagram of the study cohorts. The diagram illustrates the number of patients assessed, exclusions, and the final cohorts included in the training and validation analyses.

### **Supplemental Figure 2.** Forest plot of multivariable analysis of clinicopathology and biosignatures in women aged 50 years and older. A) Multivariable analyses for the biosignature and treatment adjusted for the clinicopathology factors, summarized for the eligible population. B) Multivariable analyses for the treatment adjusted for the clinicopathology factors, summarized for the eligible population.

### *Abbreviations: RT – radiation therapy, ET – endocrine therapy, DS – Decision Score, RRI – Radiation Resistance Index.*

###

B – Elevated Risk Group

A – Low Risk Group

###
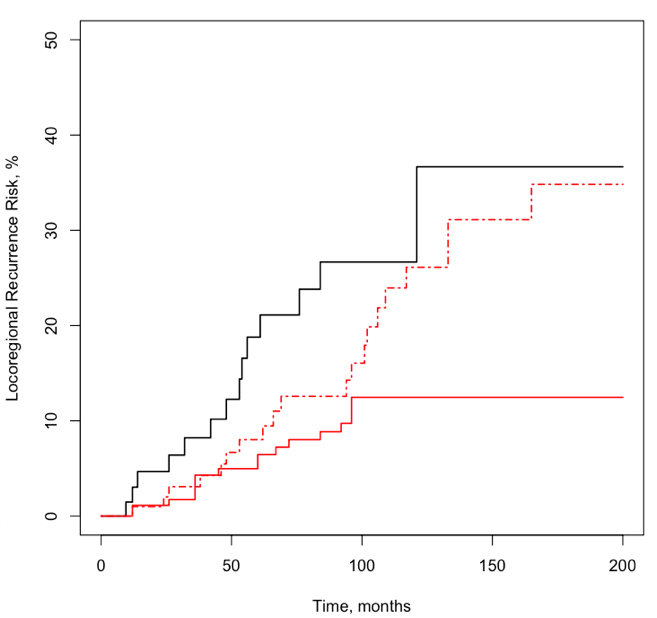


### **Supplemental Figure 3.** Kaplan Meier analysis of LRR risks by RT and biosignature groups for all ages.

### Ipsilateral breast cancer locoregional recurrence risk (LRR) over time for patients treated without RT (black-line) and those treated with RT (red-line). **A)** Subset of women classified into biosignature low risk group (DS≤5), n=559; 113 patients treated without RT and 446 treated with RT. **B)** Subset of women classified into biosignature Elevated risk group (DS>5), n=363; 73 patients treated without RT (black-line) and 290 treated with RT, where 152 treated with RT had Significant Therapeutic Benefit (STB) (RRI ≤5) (solid red-line), and 138 treated with RT had Minimal Therapeutic benefit (MTB) (RRI>5) (dot-dash red-line).

B

A

### **
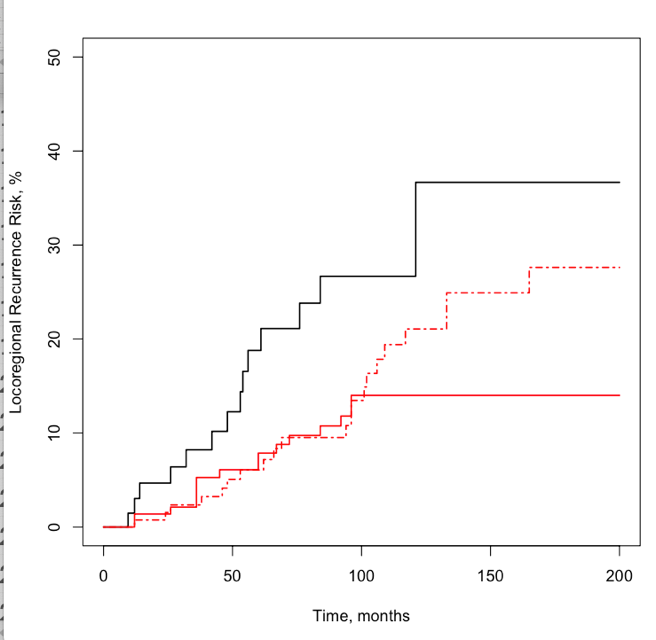
**

B- Elevated Risk Group

A-Low Risk Group

### **Supplemental Figure 4.** Kaplan Meier analysis of LRR risks by RT and biosignature groups for all ages.

### Ipsilateral breast cancer local regional recurrence risk (LRR) over time for patients treated without RT (black-lines) and those treated with RT (red-lines). **A)** Subset of women classified into biosignature low risk group (DS≤5), n=530; 107 patients treated without RT (black solid-line) and 423 treated with RT (red dash-line). **B)** Subset of women classified into biosignature Elevated risk group (DS>5), n=363; 73 patients treated without RT and 290 treated with RT, where 152 treated with RT had STB (RRI ≤3.8) (red solid-line), and 138 treated with RT had reduced RT benefit (RRI>3.8) (red dot-dash line).

###

**Supplemental Figure 5:** Ten-year LRR by categorical biosignature risk groups for women of age 50 or older 1) Low Risk (DS≤5) with RT (yellow) and without RT (blue), 2) Elevated Risk (DS>5) without RT (blue) with Significant Therapeutic Benefit (STB) (RRI≤5) (periwinkle), 2) Elevated Risk with Minimal Therapeutic Benefit (MTB) (RRI>5) (orange). HR = 4.6 (95% CI, 1.8 to 12.1), p=.0020.

**Supplemental References:**

1. Stenmark Tullberg A, Puttonen HA, Sjöström M, et al. Immune infiltrate in the primary tumor predicts effect of adjuvant radiotherapy in breast cancer; results from the randomized SweBCG91RT trial. *Clinical Cancer Research*. 2021;27(3):749-758.

2. Stenmark Tullberg A, Sjöström M, Niméus E, et al. Integrating tumor-intrinsic and Immunologic factors to identify Immunogenic breast cancers from a low-risk cohort: Results from the randomized Swebcg91Rt trial. *Clinical Cancer Research*. 2023;29(9):1783-1793.

3. Stenmark Tullberg A, Sjostrom M, Tran L, et al. Combining histological grade, TILs, and the PD-1/PD-L1 pathway to identify immunogenic tumors and de-escalate radiotherapy in early breast cancer: a secondary analysis of a randomized clinical trial. *J Immunother Cancer*. May 2023;11(5)doi:10.1136/jitc-2022-006618

4. Käll L, Storey JD, Noble WS. qvality: non-parametric estimation of q-values and posterior error probabilities. *Bioinformatics*. 2009;25(7):964-966. doi:10.1093/bioinformatics/btp021

5. Kohavi R. A study of cross-validation and bootstrap for accuracy estimation and model selection. Montreal, Canada; 1995:1137-1145.

6. Picard RR, Cook RD. Cross-Validation of Regression Models. *Journal of the American Statistical Association*. 1984/09/01 1984;79(387):575-583. doi:10.1080/01621459.1984.10478083

7. Storey JD. A Direct Approach to False Discovery Rates. *Journal of the Royal Statistical Society Series B: Statistical Methodology*. 2002;64(3):479-498. doi:10.1111/1467-9868.00346

8. Habashy HO, Powe DG, Rakha EA, et al. Forkhead-box A1 (FOXA1) expression in breast cancer and its prognostic significance. *Eur J Cancer*. Jul 2008;44(11):1541-51. doi:10.1016/j.ejca.2008.04.020

9. Shou J, Zhang Z, Lai Y, Chen Z, Huang J. Worse outcome in breast cancer with higher tumor-infiltrating FOXP3+ Tregs : a systematic review and meta-analysis. *BMC Cancer*. Aug 26 2016;16(1):687. doi:10.1186/s12885-016-2732-0

10. Harris RE, Casto BC, Harris ZM. Cyclooxygenase-2 and the inflammogenesis of breast cancer. *World J Clin Oncol*. Oct 10 2014;5(4):677-92. doi:10.5306/wjco.v5.i4.677

11. Nguyen AT, Shiao SL, McArthur HL. Advances in Combining Radiation and Immunotherapy in Breast Cancer. *Clinical Breast Cancer*. 2021;21(2):143-152. doi:10.1016/j.clbc.2021.03.007

12. Riaz N, Jeen T, Whelan TJ, Nielsen TO. Recent Advances in Optimizing Radiation Therapy Decisions in Early Invasive Breast Cancer. *Cancers (Basel)*. Feb 16 2023;15(4)doi:10.3390/cancers15041260

13. Strell C, Stenmark Tullberg A, Jetne Edelmann R, et al. Prognostic and predictive impact of stroma cells defined by PDGFRb expression in early breast cancer: results from the randomized SweBCG91RT trial. *Breast Cancer Research and Treatment*. 2021/05/01 2021;187(1):45-55. doi:10.1007/s10549-021-06136-4

14. Strell C, Folkvaljon D, Holmberg E, et al. High PDGFRb expression predicts resistance to radiotherapy in DCIS within the SweDCIS randomized trial. *Clinical Cancer Research*. 2021;27(12):3469-3477.
